# PDZK1 protects against mechanical overload-induced chondrocyte senescence and osteoarthritis by targeting mitochondrial function

**DOI:** 10.1038/s41413-024-00344-6

**Published:** 2024-07-17

**Authors:** Yan Shao, Hongbo Zhang, Hong Guan, Chunyu Wu, Weizhong Qi, Lingfeng Yang, Jianbin Yin, Haiyan Zhang, Liangliang Liu, Yuheng Lu, Yitao Zhao, Sheng Zhang, Chun Zeng, Guiqing Wang, Xiaochun Bai, Daozhang Cai

**Affiliations:** 1 Department of Joint Surgery, Center for Orthopedic Surgery, The Third Affiliated Hospital of Southern Medical University, Guangzhou, China; 2 Department of Orthopedics, Orthopedic Hospital of Guangdong Province, Academy of Orthopedics Guangdong Province, The Third Affiliated Hospital of Southern Medical University, Guangzhou, China; 3 The Third School of Clinical Medicine, Southern Medical University, Guangzhou, China; 4 Orthopedics Department, Qingyuan People’s Hospital, The Sixth Affiliated Hospital of Guangzhou Medical University, Qingyuan, Guangdong China; 5 Department of Cell Biology, School of Basic Medical Sciences, Southern Medical University, Guangzhou, Guangdong China

**Keywords:** Pathogenesis, Metabolic disorders, Bone quality and biomechanics

## Abstract

Mechanical overloading and aging are two essential factors for osteoarthritis (OA) development. Mitochondria have been identified as a mechano-transducer situated between extracellular mechanical signals and chondrocyte biology, but their roles and the associated mechanisms in mechanical stress-associated chondrocyte senescence and OA have not been elucidated. Herein, we found that PDZ domain containing 1 (PDZK1), one of the PDZ proteins, which belongs to the Na^+^/H^+^ Exchanger (NHE) regulatory factor family, is a key factor in biomechanically induced mitochondrial dysfunction and chondrocyte senescence during OA progression. PDZK1 is reduced by mechanical overload, and is diminished in the articular cartilage of OA patients, aged mice and OA mice. *Pdzk1* knockout in chondrocytes exacerbates mechanical overload-induced cartilage degeneration, whereas intraarticular injection of adeno-associated virus-expressing PDZK1 had a therapeutic effect. Moreover, PDZK1 loss impaired chondrocyte mitochondrial function with accumulated damaged mitochondria, decreased mitochondrion DNA (mtDNA) content and increased reactive oxygen species (ROS) production. PDZK1 supplementation or mitoubiquinone (MitoQ) application alleviated chondrocyte senescence and cartilage degeneration and significantly protected chondrocyte mitochondrial functions. MRNA sequencing in articular cartilage from *Pdzk1* knockout mice and controls showed that PDZK1 deficiency in chondrocytes interfered with mitochondrial function through inhibiting Hmgcs2 by increasing its ubiquitination. Our results suggested that PDZK1 deficiency plays a crucial role in mediating excessive mechanical load-induced chondrocyte senescence and is associated with mitochondrial dysfunction. PDZK1 overexpression or preservation of mitochondrial functions by MitoQ might present a new therapeutic approach for mechanical overload-induced OA.

## Introduction

Osteoarthritis (OA) is the most common degenerative joint disorder that represents a leading cause of disability and source of socioeconomic cost in older adults.^1–3^ It is recognized that mechanical overloading and advanced age are the main risk factors involved in OA pathogenesis, but the mechanisms have, to date, not been elucidated.^4–6^

In our previous study, we found that excessive mechanical overload promoted chondrocyte senescence and OA development.^7^ However, the molecular and cellular mechanisms that underlie the transduction of physical signals to biochemical signals resulting in OA progression have not been fully elucidated.^8–10^ Mitochondria, which are membrane-enclosed organelles and produce cellular energy in the form of adenosine^11^ triphosphate (ATP), are currently a focus of biomedical research because of their role in aging and the development of OA pathologies.^12,13^ Growing evidence suggests that mitochondrial dysfunction not only leads to cellular senescence, but also accelerates organismal aging via mechanisms associated with mitochondrial reactive oxygen species (ROS) production, mitochondria DNA (mtDNA) mutations and mitochondrial biogenesis.^11,14–16^ Targeted clearance of mitochondrial ROS production can significantly reduce Matrix Metallopeptidase (MMP) secretion by OA chondrocytes and delay OA cartilage degeneration.^17,18^ Recent studies have identified mitochondria as a critical mechano-transducer situated between extracellular mechanical signals and chondrocyte biology, and their function can be disordered as early as after a few minutes on mechanical overloading, leading to chondrocyte damage.^19,20^ Therefore, we propose that mitochondria play an important role in chondrocyte senescence resulting from mechanical overload, but the specific mechanisms by which mechanical stimulation leads to alteration in mitochondrial function remains to be identified.

Recently, accumulating evidence has shown that chondrocyte homeostasis can be regulated by various ion channels, which are the first to respond to mechanical stimuli.^21,22^ Although chondrocytes are non-excitable cells, they have a rich complement of ion channels, which includes potassium channels, sodium channels, transient receptor potential calcium or non-selective cation channels and chloride channels.^23^ For instance, sodium-hydrogen exchanger1 (NHE1) and NHE regulatory factor1 (NHERF1) in chondrocytes is associated with OA.^24,25^ NHERF serve as acts as a scaffold for a variety of proteins to modulate multiple NHE by forming complexes, in addition to binding cytoskeleton to perceive mechanical stress signals and transduce them into biochemical signals.^26^ Therefore, it is reasonable to hypothesis that alterations of NHERF could be associated with OA pathogenesis under mechanical overloading. Our group preliminarily examined this hypothesis by quantitative PCR (qPCR) analysis NHE and NHERF, and found that the gene encoding the PDZ domain containing 1 (PDZK1), was markedly decreased in chondrocytes treated with mechanical overloading. Because the role of PDZK1 in OA has not previously been investigated, we herein explored the possible functions and underlying molecular mechanisms of PDZK1 in OA pathogenesis. In this study, significant PDZK1 suppression was detected during chondrocyte senescence and OA progression in response to mechanical overload. *Pdzk1* knockout in chondrocytes exacerbated mechanical overload-induced cartilage degeneration, whereas supplementary PDZK1 administration had a therapeutic effect, indicating the essential role of PDZK1 in maintaining cartilage homeostasis. Moreover, PDZK1 loss impaired chondrocyte mitochondrial function through protect 3-hydroxy-3-methylglutaryl-CoA synthase 2 (Hmgcs2) from ubiquitination. Our results suggested that PDZK1 deficiency plays a crucial role in mediating excessive mechanical load-induced chondrocyte senescence and is associated with mitochondrial dysfunction.

## Results

### Chondrocyte PDZK1 is a potential candidate during chondrocyte senescence and OA progression in response to mechanical overload

**Fig. 1:**
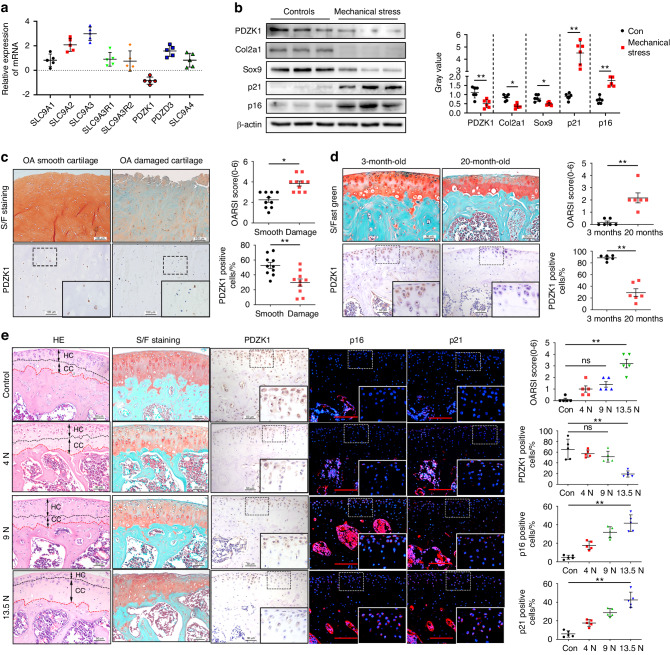
Chondrocyte PDZK1 is a potential candidate during chondrocyte senescence and OA progression responded to mechanical overload. **a** Quantitative PCR analysis of NHE and NHERF family members in mouse primary chondrocytes treated with 20% elongation strain loading for 24 h. *n* = 5. **b** Western blotting analysis and gray value of COL2A1, SOX9, PDZK1, p16^ink4a^ and p21 in primary chondrocyte suffered from 20% elongation strain loading. *n* = 6 per group. **c** Representative images of safranin O/fast green staining, IHC staining of PDZK1 and quantification analysis of OARSI scale, PDZK1-positive chondrocytes as a proportion of the total chondrocytes in smooth cartilage and damaged cartilage of OA patients. Scale bars: 100 μm. *n* = 10 per group. **d** Representative images of safranin O/fast green staining, IHC staining of PDZK1 and quantitative analysis of OARSI scale, PDZK1-positive chondrocytes as a proportion of the total chondrocytes in articular cartilage of male mice aged 3 and 20 months. Scale bars: 50 μm. *n* = 10 per group. **e** Representative images HE staining, safranin O/fast green staining, IHC staining of PDZK1, immunofluorescence of p16^ink4a^ and p21 and quantitative analysis of OARSI scale and positive chondrocytes in articular cartilage of mice treated with multiple loading episodes at peak loads of 4.0, 9.0 and 13.5 N. Scale bars: 50 μm. *n* = 5 per group Data are shown as mean + SD. ns not significant.*P < 0.05, **P < 0.01. NHE, sodium-hydrogen exchanger. NHERF NHE Regulatory Factor. IHC immunohistochemical, PDZK1 NHE Regulatory Factor 3, DMM destabilization of the medial meniscus, OA osteoarthritis, OARSI Osteoarthritis Research Society International

To investigate the role of the NHE during OA under mechanical overload, mouse primary chondrocytes were treated with 0.5 Hz and 20% cyclic tensile strain loading for 24 h and the levels of all NHE and NHERF family members were determined. PDZK1 appeared to be a promising candidate because of its specific reduction, whereas others were upregulated in response to excessive mechanical loading (Fig. 1a). Western blotting (WB) analysis of mouse primary chondrocytes confirmed a marked PDZK1 decrease by excessive mechanical loading, which had been previously proved to induce chondrocyte senescence (Fig. 1b). PDZK1 is a scaffold protein that connects plasma membrane proteins and regulatory components whose roles in chondrocytes and OA development are unknown.

Immunohistochemical (IHC) staining demonstrated a loss of PDZK1- chondrocytes in damaged cartilage that underwent excessive mechanical loading, compared to relatively normal cartilage that had not been mechanically loaded (Fig. 1c and Fig. S1A). Moreover, immunofluorescence (IF) staining showed that the senescence-related markers p16^ink4a^ and p21 were upregulated in association with inhibited PDZK1 in patients with OA (Fig. S1b). Interestingly, PDZK1 was markedly decreased in senescent chondrocytes from aged (20-month-old) mice compared to young (3-month-old) animals, as well as in degenerative cartilage that OA mice suffered following destabilization of the medial meniscus (DMM) surgery (Fig. 1d and Fig. S1c, d). We further evaluated associations for the expression level of PDZK1 and OA severity of mouse which show a positive correlation was observed between changes in PDZK1 levels and corresponding articular cartilage OARSI score (*r* = 0.701 6, *P* < 0.000 1), post-DMM surgery time points (*r* = 0.857 1, *P* < 0.000 1) (Fig. S2). Moreover, we assessed PDZK1 expression in mouse articular cartilage in response to mechanical stimulation. As expected, the application of multiple loading episodes at a peak load of 13.5 N for 14 days induced proteoglycan loss with significant fibrillation of the articular surface, whereas no obvious changes were detected on low mechanical loading of 4 N or 9 N (Fig. 1e). PDZK1-positive articular chondrocytes were progressively reduced, whereas p21 and p16^ink4a^ increased in response to increasing mechanical load. Taken together, these observations showed that chondrocyte PDZK1 is reduced by mechanical overloading and is decreased in the articular cartilage of OA patients, aged mice, and OA mice, implicating the potential role of PDZK1 to be a promising prognostic marker and therapeutic target for mechanical overload-induced OA.

### PDZK1-knockout (*Pdzk1*KO) mice exhibit exacerbated symptoms of mechanical load-induced OA

To determine the specific role of PDZK1 in OA pathophysiology, we generated *Pdzk1*KO mice using CRISPR-Cas9 (Fig. S3a). Quantitative Real-time PCR (q-PCR), WB and IHC analyses were performed to confirm that PDZK1 was efficiently deleted in the chondrocytes (Fig. S3b–e). Weight and body length assessment showed no significant phenotypic differences between the *Pdzk1*KO mice and the littermate controls (Fig. S3f). These data indicated that PDZK1 was successfully deleted in chondrocytes and the PDZK1 loss did not induce skeletal developmental abnormalities in mice.

**Fig. 2:**
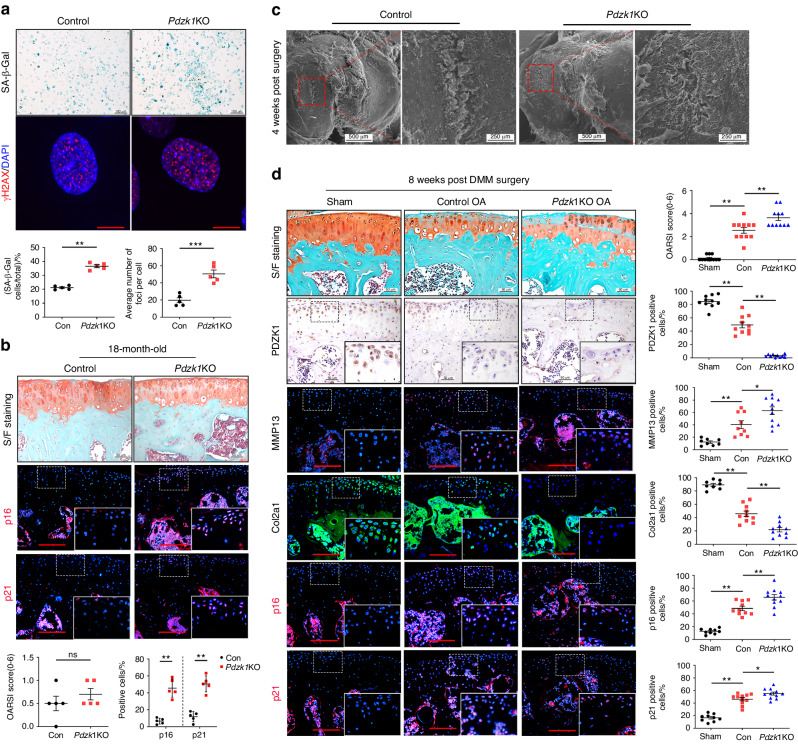
*Pdzk1*KO mice exhibit aggravated symptoms of mechanical load-induced OA. **a** Representative images of SA-β-Gal staining, immunofluorescence staining of γH2AX and quantification analysis of positive chondrocytes as a proportion of the total chondrocytes and average number of foci in passage 6 chondrocytes from *Pdzk1*KO mice and controls. Scale bars: 25 μm (SA-β-Gal) and 5 μm (γH2AX). *n* = 5 per group. **b** Representative images of safranin O/fast green staining, immunofluorescence of p16^ink4a^ and p21 and quantification analysis of OARSI scale and positive chondrocytes as a proportion of the total chondrocytes in articular cartilage of *Pdzk1*KO and Control male mice aged 18 months. Scale bars: 50 μm. *n* = 5 per group. **c** Representative scanning electron microscopy micrographs show the damage of cartilage surface. **d** Representative images of safranin O/fast green staining, IHC staining of PDZK1, MMP13, immunofluorescence of Col2a1, p16^ink4a^, p21 and quantification analysis of OARSI scale and positive chondrocytes as a proportion of the total chondrocytes in articular cartilage of *Pdzk1*KO and Controls at 8 weeks after DMM surgery and Sham group. Scale bars: 50 μm. *n* = 10 per group. Data are shown as mean + SD. ns not significant; *P < 0.05. **P < 0.01. IHC immunohistochemical, DMM destabilization of the medial meniscus, OA osteoarthritis, OARSI Osteoarthritis Research Society International

In vitro study showed that *Pdzk1* deletion promoted chondrocyte senescence because enhanced senescence-associated β-galactosidase (SA-β-Gal) staining and upregulated γH2AX, a marker of DNA damage, were observed in *Pdzk1*-deficient chondrocyte cultured at passage 6 (Fig. 2a). However, significant differences in NHE and NHERF family members were found between *Pdzk1*-deficient chondrocytes and littermate controls except SLC9A2 and SLC9A3R1(Fig. S3g). In addition, p16^ink4a^- and p21-positive chondrocytes were upregulated in 18-month-old *Pdzk1*KO mice, though no obvious cartilage damage was presented compared to controls, indicating the pro-senescence effect of PDZK1 loss in chondrocytes (Fig. 2b and Fig. S4a). We used 10-week-old male *Pdzk1*KO mice to establish a DMM OA animal model. The morphology of the tibia plateau was studied by scanning electron microscopy. A slightly stripped cartilage and superficial avulsion were observed in controls, while *Pdzk1*KO mice presented a larger area of stripped cartilage and exfoliation, and exposed subchondral bone with microcracks at 4 weeks post-OA surgery (Fig. 2c). In addition, several experimental methods were applied to study OA pathological changes under *Pdzk1* deletion. Compared to littermate controls, *Pdzk1*KO mice exhibited an accelerated loss of both proteoglycans and cellularity, leading to increased articular cartilage damage at 8 weeks post-OA surgery, which was further validated by Osteoarthritis Research Society International (OARSI) scale analysis. MMP13-positive chondrocytes were increased, whereas Collagen Type II Alpha 1 (Col2a1) and SRY-Box Transcription Factor 9 (Sox9) were significantly decreased in *Pdzk1*KO mice compared to control mice. Senescence-related markers p16^ink4a^, p21, γH2AX and p53 were significantly upregulated by PDZK1 loss (Fig. 2d and Fig. S4b). In addition, we also detected synovial inflammation and subchondral bone changes in *Pdzk1*KO mice and their littermate controls. In DMM model mice, staining scores exhibited no statistically significant difference in synovial inflammation and subchondral bone phenotype but *Pdzk1*KO mice developed more MMP13-positive cells compared with littermate controls in synovial (Fig. S5). Collectively, these results suggested that PDZK1 plays a vital role in chondrocyte senescence and OA progression, and PDZK1 deletion in chondrocytes exacerbates the mechanical overload-induced cartilage degeneration.

### PDZK1 overexpression defers chondrocyte senescence and protects against OA development

**Fig. 3:**
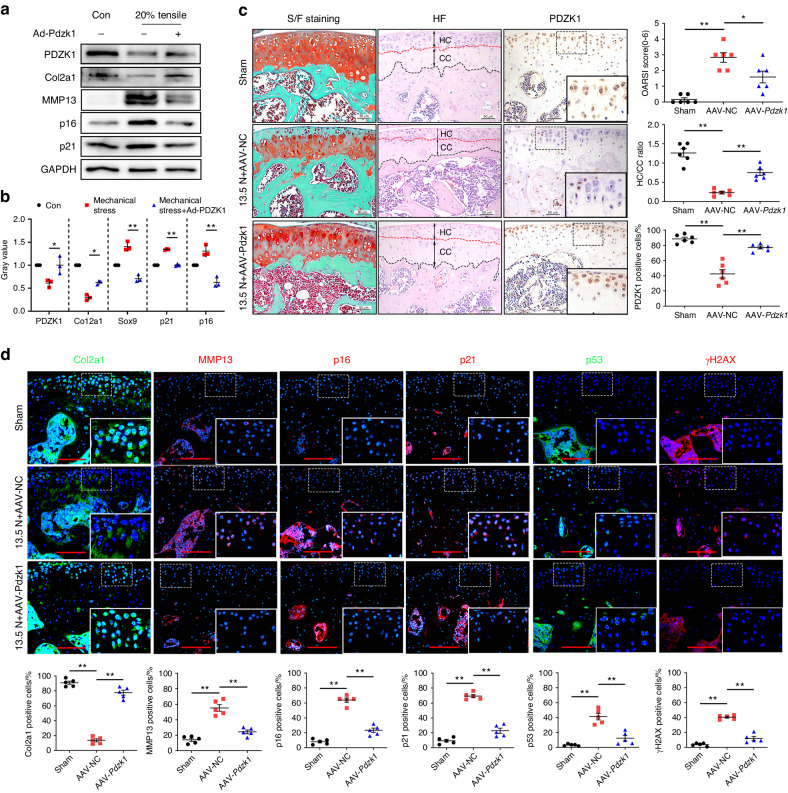
Overexpression of PDZK1 defers chondrocytes senescence and protects against OA development. **a** Western blotting analysis of COL2A1, MMP13, PDZK1, p16^ink4a^ and p21 in primary chondrocyte with or without adenovirus containing PDZK1 (Ad-Pdzk1) under 20% elongation strain loading for 24 h. **b** Gray value analysis of western blotting described in **a**. *n* = 3 per group. **c** Representative images of safranin O/fast green staining, HE staining and IHC staining of PDZK1 and quantification analysis of OARSI scale, HC/CC and PDZK1-positive chondrocytes as a proportion of the total chondrocytes in articular cartilage of PDZK1-expressing adeno-associated virus (AAV-Pdzk1) and control male mice at 4 weeks after loading of 13.5 N. Scale bars: 50 μm. *n* = 6 per group. **d** Representative images of immunofluorescence of Col2a1, MMP13, p16^ink4a^, p21, p53, γH2AX and quantitative analysis of positive chondrocytes as a proportion of the total chondrocytes in articular cartilage of AAV-Pdzk1 and control male mice at 4 weeks after loading of 13.5 N. Scale bars: 50 μm. *n* = 5 per group. ns not significant. *P < 0.05. **P < 0.01. NC negative control, IHC immunohistochemical, OA osteoarthritis, OARSI Osteoarthritis Research Society International

Subsequently, mouse primary chondrocytes were treated with or without adenovirus containing PDZK1 (Ad-Pdzk1) under 20% elongation strain loading for 24 h. It was of particular interest that the addition of PDZK1 rescued the promotion of the catabolic and senescent effects induced by excessive mechanical loading, with decreased p16^ink4a^ and p21 expression in Ad-PDZK1-treated chondrocytes (Fig. 3a and b). Subsequently, the effect of PDZK1 overexpression on OA pathogenesis was examined via intra-articular injection of PDZK1-expressing adeno-associated virus (AAV-Pdzk1) once a week from 7 days after excessive mechanical stress-induced OA surgery. IHC staining indicated that intra-articular injection of adeno-associated virus elevated PDZK1 expression mainly in articular cartilage, not in subchondral bone of AAV-Pdzk1-treated mice, demonstrating successful AAV-delivered PDZK1 overexpression (Fig. 3c). As expected, AAV-Pdzk1 effectively alleviated OA development in mice, as manifested by reduced cartilage destruction and proteoglycan loss caused by mechanical overload for 28 days, together with increased Col2a1 and decreased MMP13 expression, indicating the chondrogenesis effect of PDZK1 overexpression on OA pathogenesis (Fig. 3d). Of note, senescence-related markers p16^ink4a^, p21, γH2AX and p53 were significantly downregulated by PDZK1 supplementation (Fig. 3d). Furthermore, the protective effect against chondrocyte senescence and cartilage degeneration by PDZK1 was further verified in the DMM OA model (Fig. S6a). Collectively, these results indicated that PDZK1 plays an essential role in maintaining cartilage homeostasis, and its overexpression protects mice against OA development.

### PDZK1 deficiency in chondrocytes inhibits mitochondrial functions

**Fig. 4:**
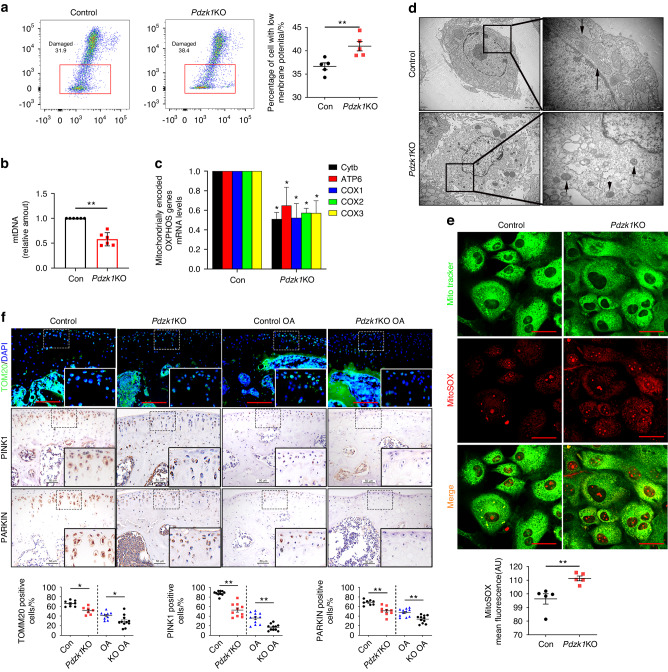
PDZK1 deficiency in chondrocytes inhibits mitochondrial functions. **a** Mitochondrial membrane potential was analyzed by flow cytometry using JC-1 dye in primary chondrocytes of *Pdzk1*KO and Control mice. Red box indicates the ratio of damaged mitochondria. *n* = 5 per group. **b** Mitochondrial DNA (mtDNA) content in chondrocytes of *Pdzk1*KO and Control mice was analyzed. Total DNA was extracted and amplified with the primer of mitochondrial cytochrome c oxidase 2 (COX2) and normalized to ribosomal protein s18 (RSP18). *n* = 6 per group. **c** Graphical representations of the relative abundance of mitochondrial‐encoded OXPHOS genes are presented. Cytb, ATP6, COX1, COX2 and COX3 mRNA levels in chondrocytes of *Pdzk1*KO and Control mice were assessed by real‐time PCR. *n* = 5 per group. **d** Transmission electron microscopy (TEM) micrographs show mitochondria ultrastructure morphology of primary chondrocytes of *Pdzk1*KO and Control mice. **e** Representative fluorescence of MitoTracker green and mitoSOX show mitochondria mass and ROS production of primary chondrocytes of *Pdzk1*KO and Control mice. Scale bars: 10 μm. Quantification analysis of mitoSOX mean fluorescence. *n* = 5 per group. **f** Representative images of immunofluorescence of TOMM20 and IHC of PINK1, PARKIN and quantitative analysis of positive chondrocytes as a proportion of the total chondrocytes in articular cartilage of *Pdzk1*KO and Controls at 8 weeks after DMM surgery and Sham group. Scale bars: 50 μm. *n* = 10 per group. ns not significant. *P < 0.05. **P < 0.01. IHC immunohistochemical, OA osteoarthritis

Previous studies have shown that excessive mechanical load can impair chondrocyte mitochondrial function during OA pathogenesis.^27^ However, the relationship between mitochondrial function and mechanically induced chondrocyte senescence and the regulatory mechanism remains unclear. Consequently, we examined the role of PDZK1 in mitochondrial function during mechanical overload-induced chondrocyte senescence and cartilage degeneration. The JC-1 staining technique was applied to detect the mitochondrial membrane potential, which has been shown to be a sensitive indicator of mitochondrial permeability.^28^ Interestingly, primary chondrocytes from *Pdzk1*KO mice displayed a significantly increased JC-1 green fluorescence when compared to controls, indicating the accumulation of damaged mitochondria in response to PDZK1 deficiency (Fig. 4a). The mtDNA content was subsequently analyzed by investigating cytochrome c oxidase subunit 2 (COX2), an important mitochondrially encoded oxidative phosphorylation (OXPHOS) complex IV subunit to transfer electrons from cytochrome c to oxygen. The mRNA expression levels of mtDNA‐encoded OXPHOS complex III subunit cytochrome b, complex V subunit ATP synthase 6, and complex IV subunits cytochrome c oxidase 1–3 (COX1, COX2 and COX3) were determined. The mtDNA content and all of these mitochondrially encoded OXPHOS genes were significantly decreased in primary chondrocytes from *Pdzk1*KO mice (Fig. 4b, c). Chondrocyte mitochondrial mass was analyzed by MitoTracker green and MitoSOX red staining, while ultrastructural analysis of mitochondria was performed by transmission electron microscopy (TEM). The MitoSOX staining showed that mitochondrial ROS production was increased in *Pdzk1*KO chondrocytes, as assessed by the mean fluorescence (Fig. 4e). In TEM analyses, normal round or rod‐shaped mitochondria and a regular arrangement of mitochondrial cristae were detected in control mice, while mitochondria became small and ovate, with little apparent cristae structure in *Pdzk1*KO mice (Fig. 4d). Additionally, there was no discernible distinction of rate-limiting enzymes associated with glucose metabolism and lipid metabolism between *Pdzk1*KO chondrocytes and littermate controls (Fig. S7). Subsequently, the outer mitochondrial membrane protein translocase of outer mitochondrial membrane 20 (TOMM20) and mitophagy markers PTEN-induced putative kinase 1 and E3 ubiquitin protein ligase (PARKIN) were assessed in *Pdzk1*KO mice and littermate controls that underwent DMM surgery. As expected, TOMM20-, PINK1- and PARKIN-positive chondrocytes were markedly decreased during OA, and further still in PDZK1-deficient chondrocytes (Fig. 4f). Overall, PDZK1 plays an important role in maintaining mitochondria intact and their natural function in chondrocytes.

### Mitoquinone (MitoQ) preserves mitochondrial functions and inhibits chondrocyte senescence induced by PDZK1 loss

**Fig. 5:**
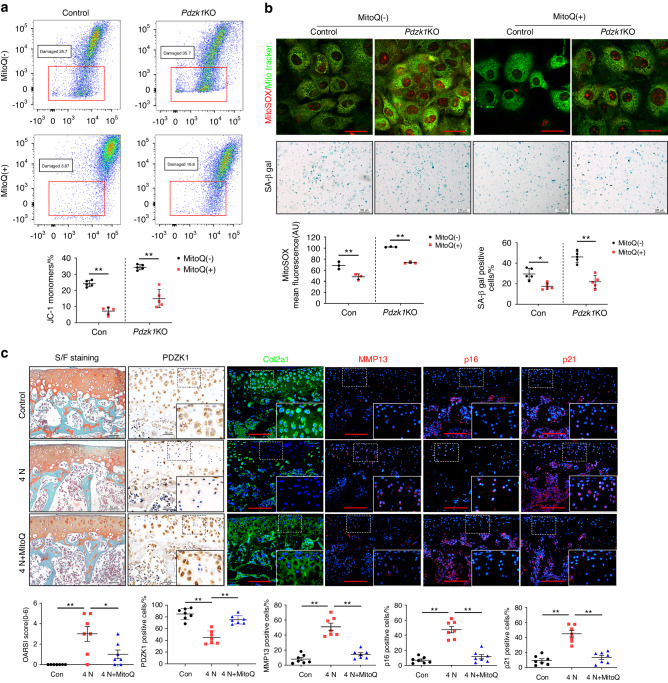
Mitoquinone (MitoQ) preserves mitochondrial functions and inhibits chondrocyte senescence induced by loss of PDZK1. **a** Mitochondrial membrane potential was analyzed by flow cytometry using JC-1 dye in passage 3 chondrocytes of *Pdzk1*KO and Control mice with or without MitoQ. Red box indicates the ratio of damaged mitochondria. *n* = 5 per group. **b** Representative fluorescence of MitoTracker green and mitoSOX and images of SA-β-Gal staining of primary chondrocytes described in **a**. Scale bar: 10 μm (MtioSOX) and 50 μm (SA-β-Gal).MitoSOX mean fluorescence and SA-β-Gal-positive chondrocytes as a proportion of the total chondrocytes were analyzed. *n* = 3 per group (MitoSOX) and *n* = 5 per group (SA-β-Gal). **c** Representative images of safranin O/fast green staining, immunofluorescence of Col2a1, MMP13, p16^ink4a^ and p21 and quantification analysis of OARSI scale and positive chondrocytes as a proportion of the total chondrocytes in cartilage explants under the application of multiple loading episodes at a peak load of 4 N for 24 h with or without MitoQ. Scale bars: 50 μm. *n* = 6 per group. *P < 0.05. **P < 0.01

To further investigate the role of PDZK1 in mitochondrial dysfunction and its association with excessive mechanical stress-induced cellular senescence, primary chondrocytes from *Pdzk1*KO mice or controls were treated at passage 3 with the mitochondria-targeted antioxidant MitoQ. Damaged mitochondria and elevated mitochondrial ROS production were rescued by MitoQ treatment in PDZK1-deficient chondrocytes, indicating that MitoQ can restore the mitochondrial function impaired by PDZK1 loss (Fig. 5b). Additionally, MitoQ inhibited chondrocyte senescence by maintaining membrane integrity (Fig. 5a). PDZK1 loss induced increased γH2AX in chondrocytes, which were significantly reduced by MitoQ, which was further confirmed by SA-β-Gal staining (Fig. 5b and online Supplemental Fig. S8). We cultured chondrocytes in 3D to simulate environment in vivo. The results showed that MitoQ remarkably decreased 3D-cultured chondrocyte senescence caused by devoid of oxygen (Fig. S9). Moreover, the protective effect of MitoQ in mechanical overload-induced cellular senescence was verified through experiments in cartilage explants from 3-week-old male mice. Significant cartilage destruction and proteoglycan loss was observed in cartilage explant under the application of multiple loading episodes at a peak load of 4 N for 24 h, together with markedly inhibited PDZK1 expression. Of note, MitoQ treatment not only alleviated cartilage destruction, but also restored chondrocyte physiological function impaired by mechanical overload, together with increasing Col2a1 and decreasing MMP13, p16^ink4a^ and p21 expression (Fig. 5c). Collectively, these results suggested that MitoQ protected against chondrocyte senescence and cartilage destruction caused by PDZK1 loss.

### PDZK1 deficiency in chondrocytes interferes with mitochondrial function through Hmgcs2

**Fig. 6:**
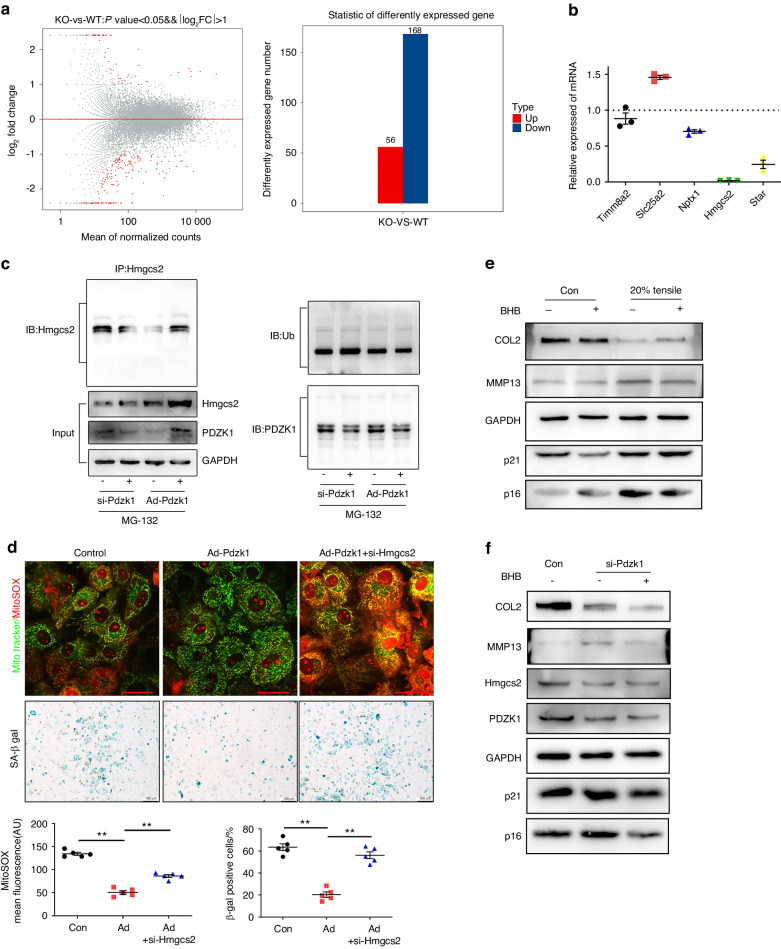
PDZK1 deficiency in chondrocytes interference with mitochondrial function through binding to Hmgcs2. **a** The mRNA expression profile of articular cartilage from *Pdzk1*KO mice and their littermate controls was analyzed. **b** Genes related to mitochondria function in *Pdzk1*KO mouse cartilage were assessed by real‐time PCR. *n* = 6 per group. **c** Hmgcs2 was immunoprecipitated from primary chondrocytes of C57 BL/6 J mice after stimulation with MG-132 and transfection with either Ad-Pdzk1 or si-Pdzk1. Western blotting detected the ubiquitination level of Hmgcs2. **d** Representative fluorescence of MitoTracker green and mitoSOX and images of SA-β-Gal staining of primary chondrocytes with or without Ad-Pdzk1 and si-Hmgcs2. Scale bar: 10 μm (MtioSOX) and 50 μm SA-β-Gal). MitoSOX mean fluorescence and SA-β-Gal-positive chondrocytes as a proportion of the total chondrocytes were analyzed. *n* = 5 per group. *P < 0.05. **P < 0.01. **e** Western blotting analysis of COL2A1, MMP13, p16^ink4a^ and p21 in primary chondrocyte suffered from 20% elongation strain loading with or without BHB. **f** Western blotting analysis of COL2A1, MMP13, PDZK1, Hmgcs2, p16^ink4a^ and p21 in primary chondrocytes treated with si-Pdzk1 or BHB. IHC immunohistochemical, OA osteoarthritis, DMM destabilization of the medial meniscus, Ad-Pdzk1 adenovirus containing PDZK1, BHB β-hydroxybutyric

To investigate the mechanisms through which PDZK1 regulates mitochondrial function and chondrocyte senescence, the mRNA expression profile of articular cartilage from *Pdzk1*KO mice and their littermate controls was analyzed (Fig. 6a). By performing Gene Ontology and Kyoto Encyclopedia of Genes and Genomes analysis, we found that genes associated mitochondrial function were considerably abated in *Pdzk1*KO mouse cartilage, and Hmgcs2 (3-Hydroxy-3-Methylglutaryl-CoA Synthase 2) was the most highly downregulated gene induced by excessive mechanical stress (Fig. 6b). Because PDZK1 can influence protein expression by preventing its ubiquitination,^29^ we examined the effect of PDZK1 silencing and overexpression on the levels of Hmgcs2 ubiquitination in the presence of the proteasome inhibitor MG132. Results showed that PDZK1 associated with Hmgcs2, and medicated Hmgcs2 ubiquitination and degradation by proteasomes (Fig. 6c). Hmgcs2 is a mitochondrial enzyme that catalyzes the first reaction of ketogenesis, which includes acetoacetic acid, β-hydroxybutyric(BHB) acid and acetone. IHC staining showed that Hmgcs2 was expressed abundantly in normal chondrocytes, whereas it was significantly decreased during OA and in PDZK1-deficient chondrocytes (Fig. S10a–d). Importantly, the protective effect of PDZK1 against mitochondrial dysfunction and chondrocyte senescence induced by excessive mechanical stress was further diminished by silencing Hmgcs2, as confirmed by the detection of elevated senescence markers γH2AX, SA-β-Gal and MitoSOX staining (Fig. 6d and Fig. S10e). Besides, mitochondrial functions were turned upside down by silencing Hmgcs2 of primary chondrocytes (Fig. S11). In addition, we further deciphered the effects of BHB on chondrocyte senescence and cartilage homeostasis using mouse primary chondrocytes incubated with either mechanical stress or PDZK1 siRNA. Results showed that BHB rescued the promotion of the catabolic and senescent effects induced by excessive mechanical loading or siRNA, with decreased p16^ink4a^ and p21 expression in BHB-treated chondrocytes (Fig. 6e, f). Taken together, the above findings indicated that PDZK1 loss may promote chondrocyte mitochondrial dysfunction and senescence through Hmgcs2.

## Discussion

**Fig. 7:**
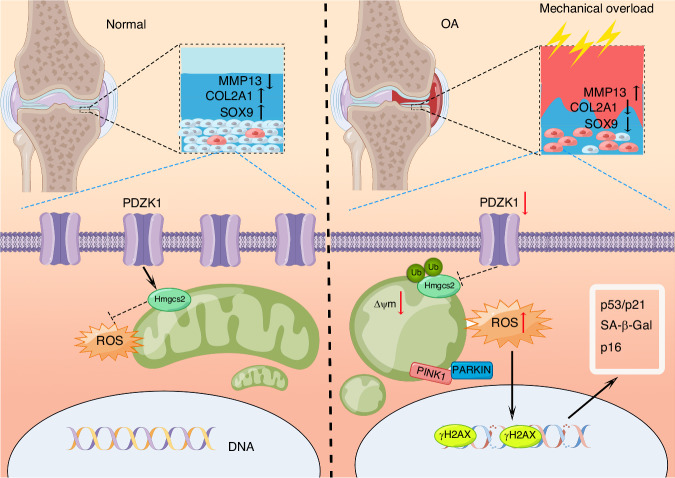
PDZK1 protects against mechanical overload-induced chondrocyte senescence and osteoarthritis via targeting mitochondrial function

In the present study, we identified PDZK1 as a key factor in biomechanically-induced mitochondrial dysfunction and chondrocyte senescence during OA progression. We showed that PDZK1 expression was markedly downregulated by mechanical overload, which in turn impaired chondrocyte mitochondrial functions through inhibiting Hmgcs2 posttranslationally by increasing its ubiquitination, consequently exacerbating chondrocyte senescence and cartilage degeneration (Fig. 7). PDZK1 supplementation or preservation of mitochondrial functions is thus a potential therapeutic target for OA treatment.

Mechanical overloading and aging are two essential factors for OA. We previously proved that excessive mechanical loading stimulates chondrocyte senescence and consequently initiates and accelerates OA development, but the mechanism is not completely understood.^7^ The mitochondrion, an energy factory that converts nutritional molecules into ATP via oxidative phosphorylation, has been identified as a mechano-transducer situated between extracellular mechanical signals and cell biology.^27,30^ It has been implied that mechanical stress affects mitochondrial ATP production and induces membrane potential change and increased oxytoxin generation and release, which act as a causal link between mitochondrial dysfunction and OA pathogenesis.^31,32^ However, the role and associated mechanism of the mitochondrion in mechanical stress-associated chondrocyte senescence and OA are not elucidated. Therefore, a better understanding of mitochondrial function would be of great significance to ameliorate aging and delay OA progression.

PDZK1 is a member of the PDZ protein family, contains four PDZ domains, can bind to various proteins through its PDZ domain, and participates in tumor formation and development.^33,34^ It is involved in regulating multiple signal transduction pathways, which have the functions of protein regulation, membrane protein modification, and playing an important role in ion transport, protein localization, cell proliferation and differentiation, and intercellular interaction.^35,36^ Although PDZK1 has been widely studied, its role in mitochondrial function and chondrocyte senescence has not been reported. In the current study, we found that PDZK1 was markedly downregulated in chondrocytes on excessive mechanical loading in vitro and in vivo. PDZK1 deletion in chondrocytes resulted in a decreased number of mitochondria and abnormal structure associated with mitochondrial dysfunction, which contributes to chondrocyte senescence and articular cartilage degeneration and consequently exacerbation of OA. In addition, we detected senescent chondrocytes in articular cartilage of DMM-OA mice and on mechanically overloading mice, which was exacerbated by PDZK1 deletion and reversed by PDZK1 overexpression or MitoQ supplementation. Therefore, we propose that mechanical overloading reduces PDZK1 expression in chondrocytes, which represents a novel mechanism for mitochondrial dysfunction and chondrocyte senescence during OA.

To identify the PDZK1 downstream mechanisms during chondrocyte senescence and OA development, we demonstrated the protein interaction of PDZK1 with Hmgcs2, which was downregulated in chondrocytes after mechanical overloading. The protein encoded by *Hmgcs2*, as a member of the HMG-CoA synthase family, is a kind of mitochondrial enzyme that catalyzes the first reaction of ketogenesis, a metabolic pathway that provides lipid-derived energy for various organs during times of carbohydrate deprivation.^37,38^ However, the role of Hmgcs2 in maintaining mitochondria function and OA pathogenesis has not been studied. We found that Hmgcs2 participated in mechanical overloading-induced mitochondrial dysfunction and was downregulated in the articular cartilage of OA and *Pdzk1*KO mice. In the present study, we found that PDZK1 inhibition by excessive mechanical stress strongly decreased Hmgcs2 expression and subsequently induced ROS accumulation, resulting in enhanced cell senescence. Additionally, the protective effect of PDZK1 by Ad-*Pdzk1* can be diminished by silencing Hmgcs2. These results indicated that the PDZK1-Hmgcs2 pathway plays an important role in mechanical stress-induced chondrocyte mitochondrial dysfunction and senescence.

In conclusion, our study found that PDZK1 deficiency-induced mitochondrial dysfunction plays an essential role in mechanical stress-associated chondrocyte senescence and OA. Overexpression of PDZK1, its the preservation of mitochondrial functions by MitoQ, or supplement BHB might present a new therapeutic approach for mechanical overload-induced OA.

## Materials and methods

### Human OA specimens

Human tibia plateaus were obtained from OA patients undergoing total knee replacement surgery who had signed informed consents. Specimens from osteoarthritic cartilage were classified as relatively smooth cartilage or severely damaged cartilage (Fig. S1a). Patients with malignant tumors, diabetes, or other serious chronic diseases were excluded. All samples were obtained from the Department of Orthopedics, The Third Affiliated Hospital of Southern Medical University.

### Primary chondrocyte isolation and culture

Primary chondrocytes were isolated from C57BL/6 J and *Pdzk1*KO mice (1-week-old). Specifically, cartilage tissues were obtained from the tibial plateau and first digested for 30 min in 0.25% trypsin-EDTA at 37 °C. Cartilage tissues were cut into 1–2 mm pieces and digested in 0.2% collagenase II for 6 h at 37 °C in a constant-temperature shaker (Hecheng, Shanghai, China). Subsequently, chondrocytes were collected and resuspended in DMEM/F12 supplemented with 10% fetal bovine serum and 1% penicillin-streptomycin (Corning, USA).

### 3D culture

To mimic the environment in vivo, we cultured the chondrocytes in the VitroGel® RGD High Concentration hydrogel (The Well Bioscience, TWG003). We adjusted the concentration of VitroGel by diluting the VitroGel with VitroGel Dilution Solution. After dilution, we gently mixed the diluted VitroGel with a cell suspension of 2 × 10^6^ cells/mL without introducing bubbles. The hydrogel mixture were transferred to 6-well plate and waited 20 min at room temperature for a soft gel formation. Then we covered cover hydrogel with additional medium to further stabilize the hydrogel and changed cover medium every 48 h.

### In vitro mechanical overloading experiment

Aberrant mechanical force was performed using a Flexcell FX-5000 tension system (Flexcell International Corp, USA), as previously described.^7^ Primary chondrocytes in the present study were treated with 20% tension intensity at 0.5 Hz for 24 h and collected for further analyses.

### Experimental OA and histological analyses

All C57BL/6 J male mice were purchased from the Laboratory Animal Center of Southern Medical University (Guangzhou, China). Experimental OA was induced by DMM surgery using 10-week-old male mice.^39^ Cartilages were processed for histological and biochemical analyses. Knee joints were fixed in 4% paraformaldehyde for 24 h, decalcified in 10% EDTA (pH 7.4) for 21 days (replenished every 3 days), dehydrated and embedded in paraffin. The paraffin blocks were sectioned at 4-µm thickness and sectioned joints were stained by safranin O and HE staining. The degree of cartilage degradation was scored at grades 0–6 using the OARSI scoring system and evaluated by the ratio of hyaline cartilage layer thickness to calcified cartilage layer thickness. The images were acquired using a ZEISS Axio Scope A1 (ZEISS, German). For Krenn’s synovitis inflammation scoring system, safranin O slides were to evaluate by scoring enlargement of the synovial lining cell layer, density of the resident cells, and inflammatory infiltrate, graded separately (from 0 to 3). For subchondral bone grading, safranin O slides were to evaluate by subchondral bone sclerosis, subchondral bone plate thickness and trabeculae grading four-stage.

### In vivo mechanical overload animal model

Right knee joints of mice were subjected to 60 cycles of 4 N, 9 N or 13.5 N axial compressive loads at 0.1 Hz twice a week to perform mechanical overload-induced OA with the Electro Force 5100 (USA). Control mice were placed within the test frame and their knees were subjected to 10 min of a static axial compressive load at 0.5 N. After 4 weeks, mice were euthanized for the collection of knee-joint specimens. Subsequently, the samples were fixed, decalcified, embedded and sectioned. The Southern Medical University Animal Care and Use Committee approved all procedures involving mice.

### Generation of *Pdzk1*KO mice

Global *Pdzk1*KO mice were generated by germline transmission of an RGEN-induced mutant allele. PDZK1-specific guide RNA and Cas9 protein (each 50 ng/μL) were injected into the cytoplasm of C57BL/6 J mouse eggs and transferred into the pseudo-pregnant foster female mice. The absence of PDZK1 in the mutants was confirmed by routine tail DNA genotyping and WB. The absence of PDZK1 was confirmed by standard PCR genotyping (Fig. S2).

### Cartilage explants

Three-week-old male C57 mice were euthanized to isolate tibial plateau cartilage explants. A stereo microscope (Olympus, Tokyo, Japan) was used to blunt dissect cartilage from underlying bone. Explants were cultured for 3 days in DMEM/F12 containing 10% fetal bovine serum and 1% penicillin-streptomycin in six-well plates (Corning) before further processing. The explants were subjected to 4 N compressive loads at 0.1 Hz for 24 h in BioDynamic instrument with the Electro Force 5100 (USA).

### Intra-articular delivery of PDZK1 overexpression adeno-associated virus (AAV-Pdzk1) in experimental OA

Overexpressing adeno-associated virus (AAV-Pdzk1) (IgeBio, GuangZhou, China) was administered to C57BL/6 J male mice with DMM- or mechanical stress-induced OA by intra-articular injection performed once a week post DMM surgery and mechanical stress-induced OA. The control groups were all treated with negative control (AAV-NC) for the same periods.

### Immunohistochemistry and immunofluorescence

Mouse and human knee cartilage samples were processed for histology as described previously.^7^ For IHC, sections were digested with Tris-EDTA buffer at pH 9 for 6 h at 60 °C and incubated overnight at 4 °C with primary antibody. Horseradish peroxidase (HRP)-conjugated antibody (Jackson ImmunoResearch Laboratories, Inc., West Grove, PA, USA) was used as a secondary antibody and staining substrate, which was stained with DAB substrate kit (cat# DAB057). For IF, sections were stained with Alexa 488 or Alexa 594 dye-labeled secondary antibodies (Invitrogen, Thermo Fisher Scientific, USA). Nuclei were labeled with 4, 6-diamidino-2-phenylindole (Sigma, USA) and images were obtained using a FluoView FV1000 confocal microscope (Olympus). Sections were randomly coded and scored by two blinded observers for three sections per joint.

### Transmission electron microscopy

To assess mitochondrial morphology, primary chondrocytes were collected and fixed in 2.5% glutaraldehyde/0.1 mol/L phosphate buffer. Ultrathin sections (60 nm) were prepared by a routine procedure^40^ and examined under an electron microscope (HITACHI HT7800).

### Scanning electron microscopy

To assess articular cartilage surface structure, right legs were collected and fixed in 2.5% glutaraldehyde/0.1 mol/L phosphate buffer at 4 °C overnight. The samples were rinsed three times with 0.1 mol/L, pH 7.0 phosphate buffer for 15 min each time, fixed in 1% osmic acid solution for 1 h and the osmic acid waste solution was carefully removed. The samples were rinsed three times with 0.1 mol/L, pH 7.0 phosphate buffer for 15 min each time, dehydrated using a gradient concentration (including 50%, 70%, 80%, 90% and 95% five concentrations) of ethanol solutions for 15 min at each concentration, and finally treated with 100% ethanol twice, both times for 20 min. The samples were subsequently treated with a mixture of ethanol and isoamyl acetate (V/V = 1/1) for 30 min followed by pure isoamyl acetate for 1 h or left overnight. Critical point drying was performed and the sections were coated and observed using a scanning electron microscope (HITACHI SU8010).

### MitoTracker and MitoSOX staining

Mitochondrial superoxide was detected using the fluorescent MitoSOX probe (Invitrogen) and the fluorescent MitoTracker probe (Yeasen, Shanghai, China). Cells were incubated in Hank’s buffer with 2 μmol/L MitoSOX-Red and 2 μmol/L MitoTracker-Green for 30 min at 37 °C in a 5% CO_2_ atmosphere, washed with phosphate-buffered saline (PBS) and assessed by confocal microscopy (Olympus FV1200).

### Analysis of mitochondrial membrane potential by flow cytometry

Following digestion with 0.25% trypsin, the cells were observed to become round under the microscope. Serum-containing medium was added to stop the digestion, the cell-containing fluid was collected and centrifuged at 1 000 r/min for 5 min, the supernatant was discarded and 1 mL DMEM/F12 was added to each tube. The cells were resuspend in clear basal medium, while an appropriate amount of JC-1 (200 μmol/L) was warmed to room temperature, of which 10 μL was added to the medium in each well to a final concentration of 2 μmol/L, shaken, mixed well and incubated for 15–20 min at 37 °C. The supernatant was removed by suction and the cells washed twice with PBS. After adding 500 μL PBS, the samples were analyzed by flow cytometry (green fluorescence: Ex/Em = 510/527 nm; red fluorescence: Ex/Em = 585/590 nm) using FlowJo 7.6 software.

### SA-β-Gal assay

The primary chondrocytes of *Pdzk1*KO mice and control mice were stained using the SA-β-Gal staining kit (Biovision, Milpitas, CA, USA) according to the manufacturer’s protocol. Following overnight staining at 37 °C, senescent chondrocytes were dyed blue. The positive cells were counted in four randomly selected fields per treatment (*n* = 5).

### Measurement of mitochondria mtDNA

MtDNA was isolated from chondrocytes using genomic and mtDNA isolation kits according to the manufacturer’s protocol (BioVision Research Products, Mountain View, CA, USA). MtDNA was amplified using primers for the *Cox2* gene and normalized to ribosomal protein s18. The sequences of the primer pairs are shown in Table S1.

### Western blotting analysis

Tissues and cells were lysed using RIPA buffer (Keygen, China) at 0 °C for 10 min. Samples were separated by SDS-PAGE for 90 min, blotted onto nitrocellulose membranes for 2 h and incubated with primary antibodies (in 5% bovine serum albumin, 0.2% NaN_3_) at 4 °C overnight. Samples were incubated with secondary antibodies at 37 °C for 1 h. The proteins were detected using an enhanced chemiluminescence kit (ECL kit, Fdbio, China).

### RNA isolation and quantitative Reverse Transcription-Polymerase Chain Reaction (RT-PCR)

Total RNA was isolated from primary chondrocytes and cartilage of mice with TRIzol reagent (Takara Bio Inc, Shiga, Japan). For mRNA quantification, 1 mg total RNA was purified with genomic DNA remover and reverse transcribed using 5× HiScript II qRT Super-Mix II (Vazyme Biotech, Nanjing, China). Each PCR reaction consisted of 10 μL 2× ChamQ SYBR qPCR Master Mix (Vazyme), 10 μmol/L forward and reverse primers, and 500 ng cDNA on a light cycler (Roche). Each gene was normalized to the expression level of the housekeeping gene *Gapdh*. The sequences of the primer pairs are shown in Table S1.

### Antibodies

The following antibodies were used: rabbit anti-PDZK1 (1:1 000 for WB, 1:200 for IF and IHC; ProteinTech, USA), rabbit anti-γH2AX (1:100 for IF; Cell Signaling Technology), rabbit anti-MMP13 (1:200 for IF, Abcam), rabbit anti-collagen II (1:200 for IF, Abcam), mouse anti-p16^ink4a^ (1:1 000 for WB, 1:200 for IF; Abcam), rabbit anti-p21 (1:2 000 for WB, 1:200 for IF; Abcam), mouse anti-GAPDH (1:3 000 for WB; ProteinTech), anti-rabbit IgG light chain (1:2 000 for WB; Abcam), HRP-labeled goat anti-rabbit IgG H&L (1:3 000 for WB, 1:200 for IHC; Abcam), HRP-labeled goat anti-mouse IgG H&L (1:3 000 for WB; Abcam), Alexa-Fluor-594-labeled goat anti-rabbit IgG H&L (1:800 for IF; Thermo Fisher Scientific), Alexa-Fluor-488-labeled goat anti-rabbit IgG H&L (1:800 for IF; Thermo Fisher Scientific), Alexa-Fluor-594-labeled goat anti-mouse IgG H&L (1:800 for IF; Thermo Fisher Scientific) and Alexa-Fluor-488-labeled goat anti-mouse IgG H&L (1:800 for IF; Thermo Fisher Scientific).

### Statistical analysis

All experiments were performed at least three times. All data are presented as the means ± SD using the SPSS version 20.0 software, and graphs were generated with GraphPad Prism 8.0. Differences between two groups were analyzed using Student’s *t*-tests. For comparison among more than two groups, one-way analysis of variance (ANOVA) followed by Tukey’s or Dunnett’s multiple comparison test was performed. For comparisons among multiple groups and different time points, two-way ANOVA followed by Sidak’s or Dunnett’s multiple comparisons test was applied. Values of *P* < 0.05 were considered to be statistically significant. The experiments were randomized, and the investigators were blinded to allocation during experiments and outcome assessment.

### Supplementary information

Supplementary information

## Data Availability

All data generated or analyzed during this study are included in this submitted article and its additional files. The mRNA sequencing data has been deposited in the NCBlSRA database under accession codes PRINA1080460.
