# Supplementary material for: PDZK1 protects against mechanical overload-induced chondrocyte senescence and osteoarthritis by targeting mitochondrial function
Source: Bone Res. 2024 Jul 17;12:41. doi: 10.1038/s41413-024-00344-6 (PMC11255281; doi:10.1038/s41413-024-00344-6)
Supplement: Supplementary file 1 — Supplementary information [file 41413_2024_344_MOESM1_ESM.docx]

**Supplementary information**


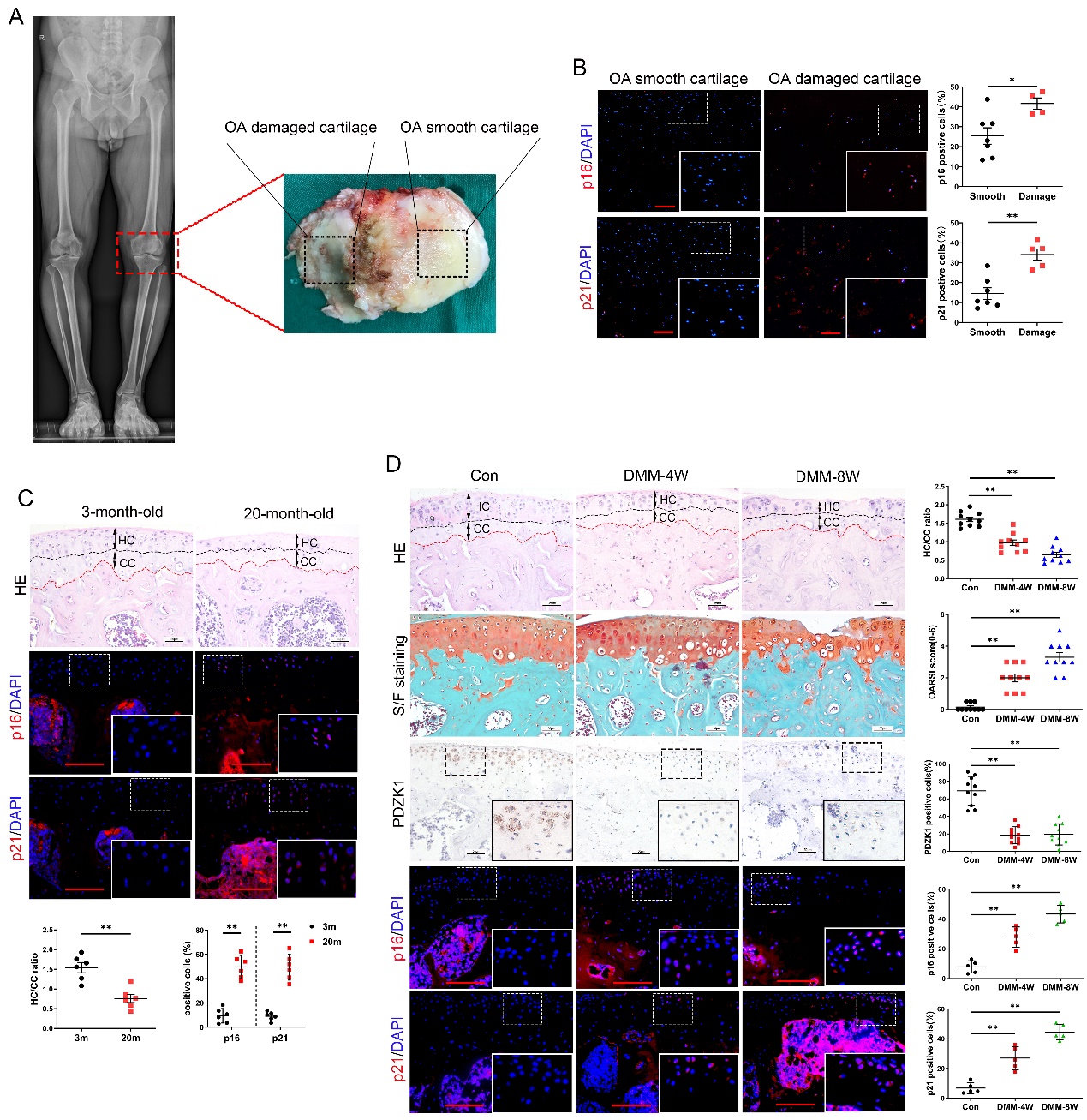


Supplementary figure 1 (A) Representative images of X-ray and tibial plateau of OA patients. Black box indicates OA smooth cartilage and damaged cartilage. (B) Representative images of immunofluorescence of p16^INK4a^ and p21 and quantification analysis of positive chondrocytes as a proportion of the total chondrocytes in smooth cartilage and damaged cartilage of OA patients. Scale bars: 100μm. n≥5 per group. (C) Representative images of HE staining, immunofluorescence of p16INK4a, p21 and quantitative analysis of HC/CC ratio, p16^INK4a^, p21-positive chondrocytes as a proportion of the total chondrocytes in articular cartilage of mice aged 3 and 20 months. Scale bars: 50μm. n=6 per group. (D) Representative images of HE staining, safranin O/fast green staining, IHC staining of PDZK1, immunofluorescence of p16^ink4a^ and p21 and quantitative analysis of OARSI scale and positive chondrocytes in articular cartilage of control and DMM mice. Scale bars: 50μm. n≥5 per group. *P<0.05. **P<0.01. IHC, immunohistochemical; PDZK1, NHE Regulatory Factor 3; DMM, destabilization of the medial meniscus; OA, osteoarthritis; OARSI, Osteoarthritis Research Society International; HC/CC, hyaline cartilage divided by calcified cartilage.


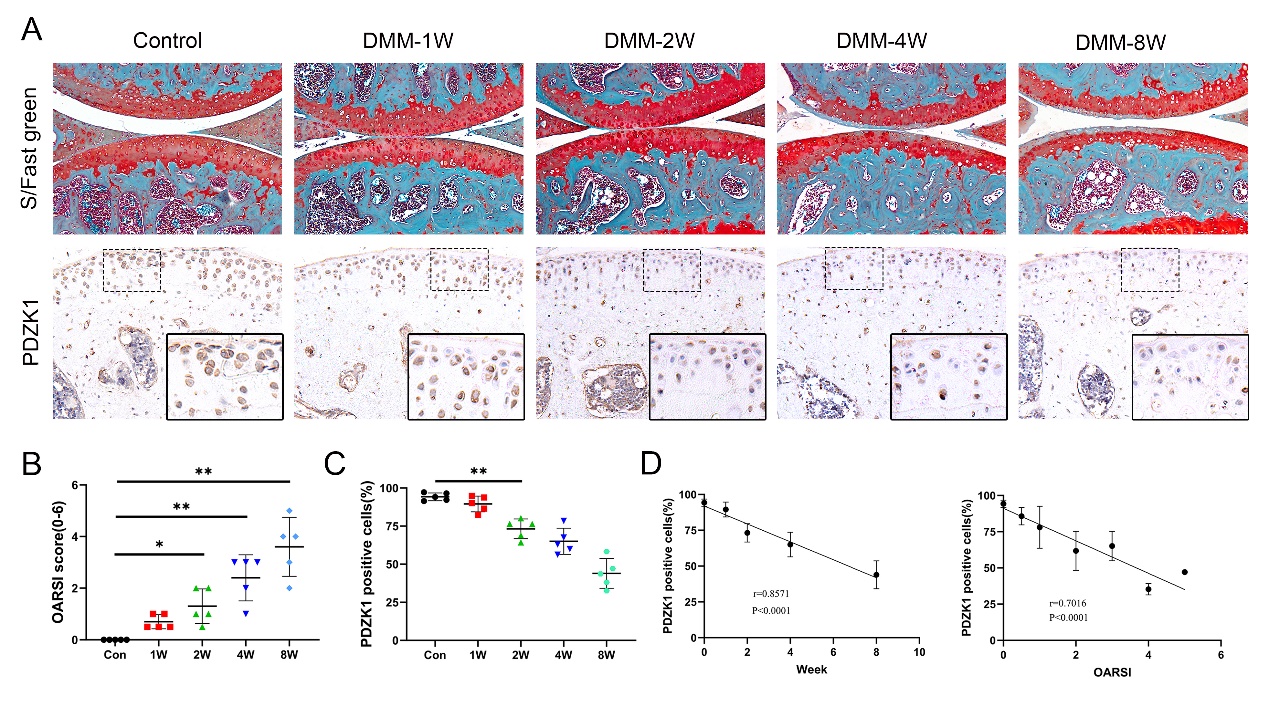


Supplementary figure 2 (A) Representative images of safranin O/fast green staining and IHC staining of PDZK1 in articular cartilage of control and DMM mice which were taken samples at different times. (B-C) Quantitative analysis of OARSI scale and PDZK1 positive chondrocytes in articular cartilage of control and DMM mice. (D) The spearman correlation analysis between OA severity and the expression level of PDZK1. n=5 per group.


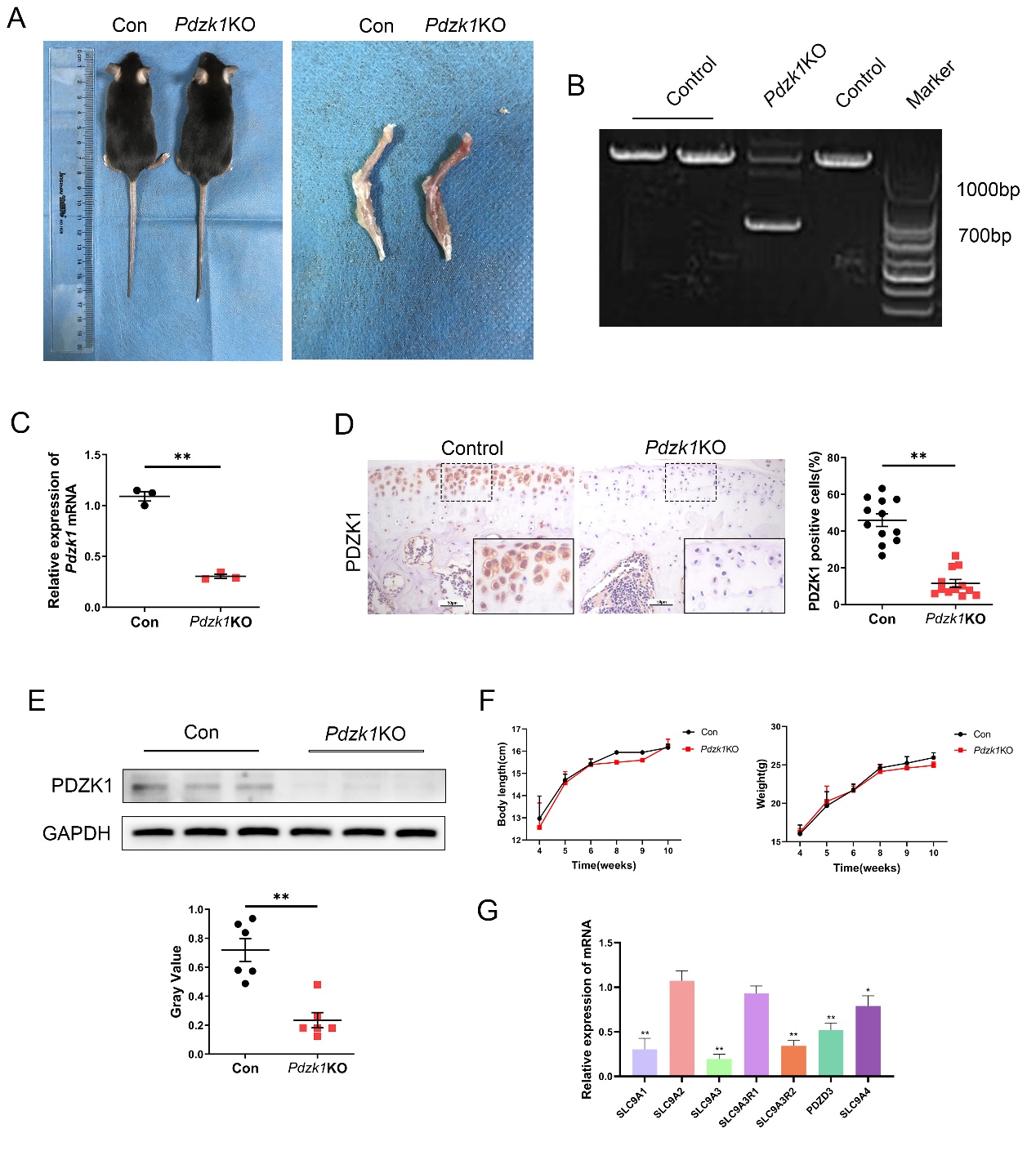


Supplementary figure 3 (A) Representative images of whole body and lower body of *Pdzk1*KO and Control mice aged 10 weeks. (B) Representative image of gel electrophoresis of *Pdzk1*KO mice genetic identification after PCR. (C) PDZK1 in the primary chondrocyte of *Pdzk1*KO and control mice were assessed by real‐time PCR. n=3 per group. (D) Representative images of IHC staining of PDZK1 and quantitative analysis of PDZK1-positive chondrocytes as a proportion of the total chondrocytes in articular cartilage of control and *Pdzk1*KO mice aged 12 weeks. Scale bars: 50μm. n=12 per group. (E) Western blotting analysis and gray value of PDZK1 in primary chondrocytes of *Pdzk1*KO and control mice. n=6 per group. (F) Quantitative analysis of weight and body length of *Pdzk1*KO and control mice aged different weeks. n=3 per group. NS, not significant. *P<0.05. **P<0.01. IHC, immunohistochemical. (G) NHE and NHERF family members in the primary chondrocytes of *Pdzk1*KO and control mice were assessed by real-time PCR. n=3 per group.


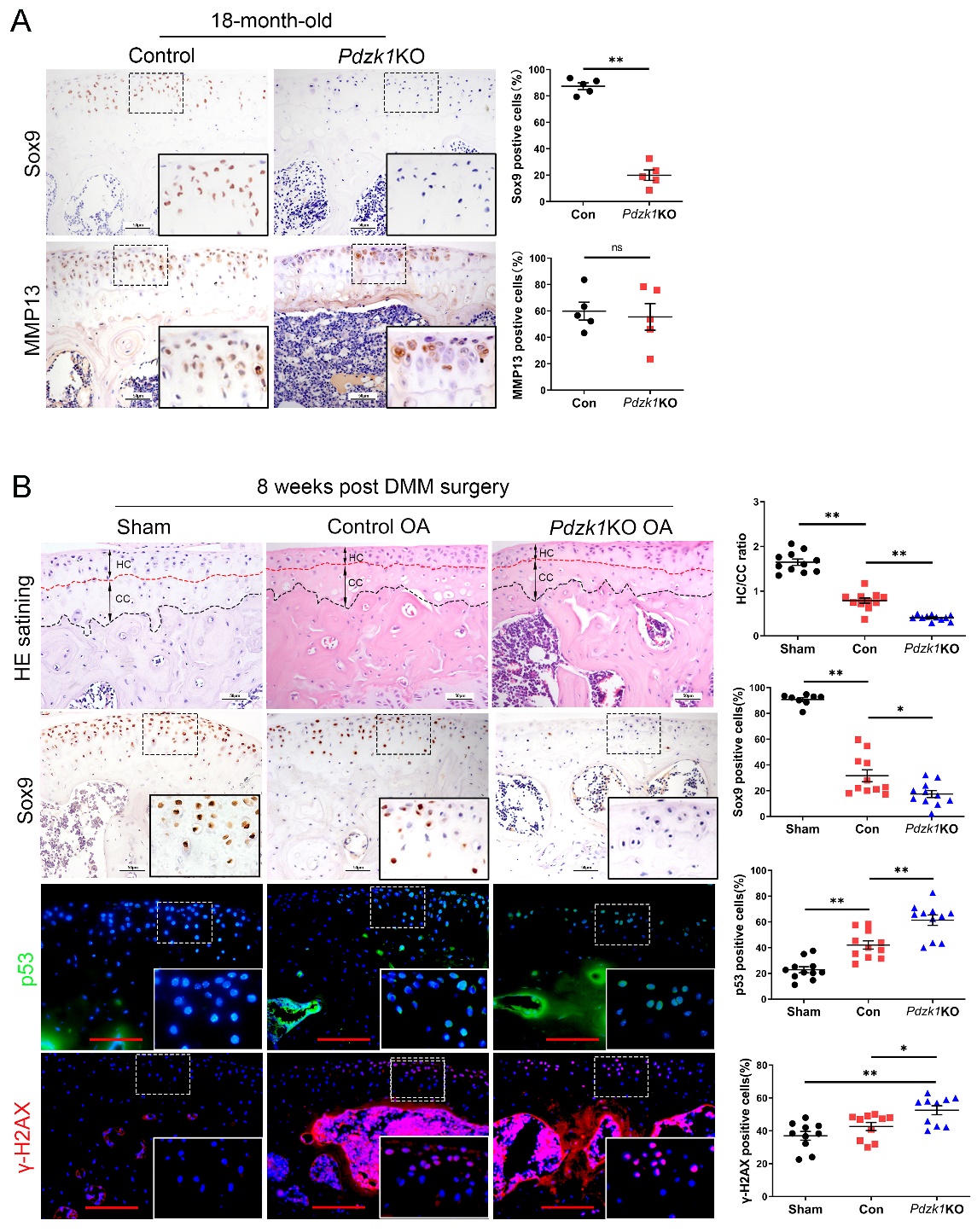


Supplementary figure 4 (A) Representative images of IHC of SOX9 and MMP13 and quantitative analysis of positive chondrocytes as a proportion of the total chondrocytes in articular cartilage of control and *Pdzk1*KO mice aged 18 months. Scale bars: 50μm. n=5 per group. (B) Representative images of HE staining, IHC of SOX9, immunofluorescence of p53 and γH2AX and quantitative analysis of HC/CC ratio and positive chondrocytes as a proportion of the total chondrocytes in articular cartilage of *Pdzk1*KO and Controls at 8 weeks after DMM surgery and Sham group. Scale bars: 50μm. n=10 per group. NS, not significant. *P<0.05. **P<0.01. IHC, immunohistochemical; HC/CC, hyaline cartilage divided by calcified cartilage.


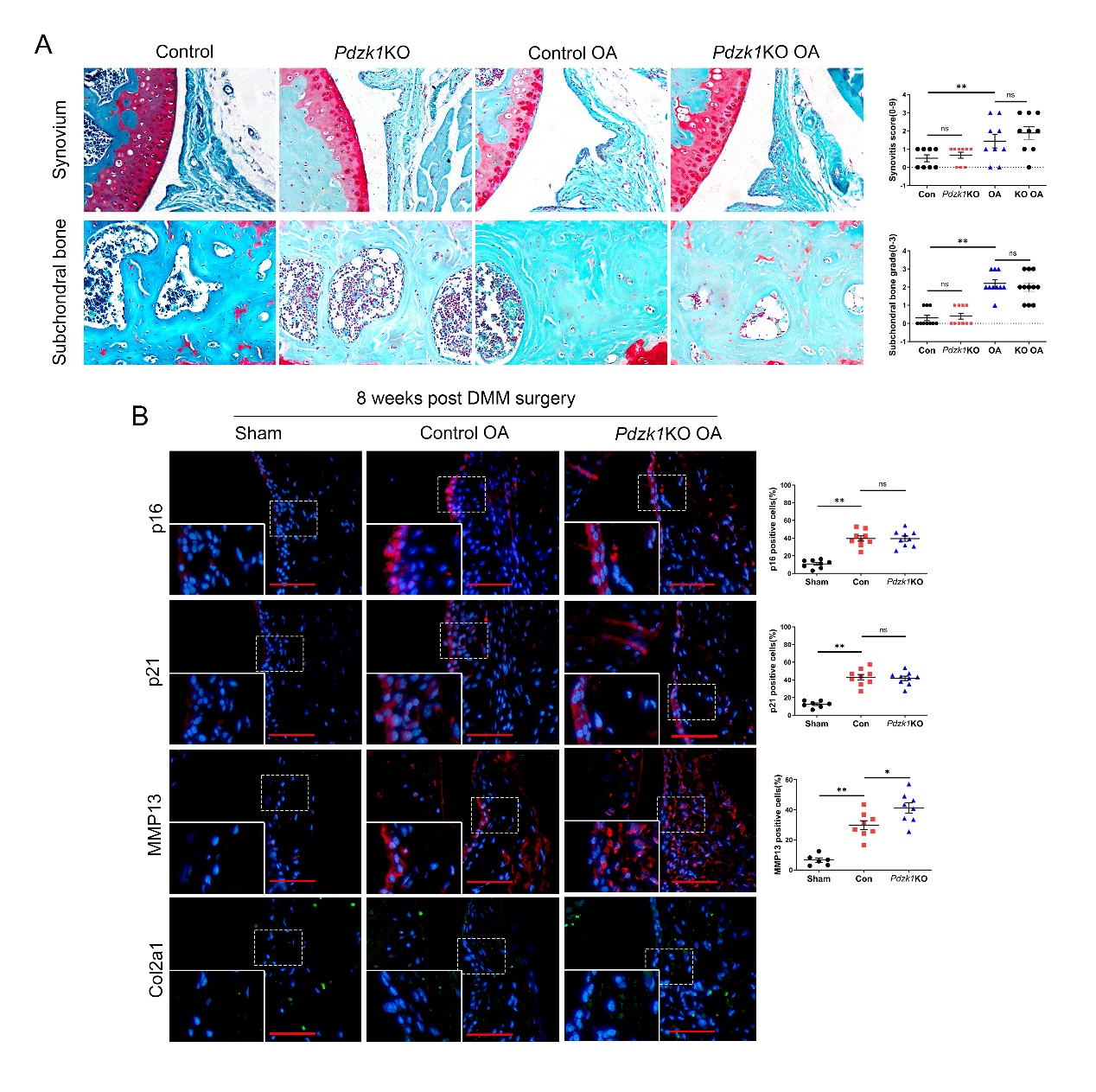


Supplementary figure 5 (A) Representative images of safranin O/fast green staining and quantitative analysis of synovial inflammation and sunchondral bone phenotype scoring in *Pdzk1*KO and Controls at 8 weeks after DMM surgery and Sham group. Scale bars: 50μm. n=10 per group. (B) Representative images of immunofluorescence of p16^INK4a^, p21,Col2a1 and MMP13 and positive chondrocytes as a proportion of the total chondrocytes in articular cartilage of *Pdzk1*KO and Controls at 8 weeks after DMM surgery. Scale bars: 50μm. n=10 per group.


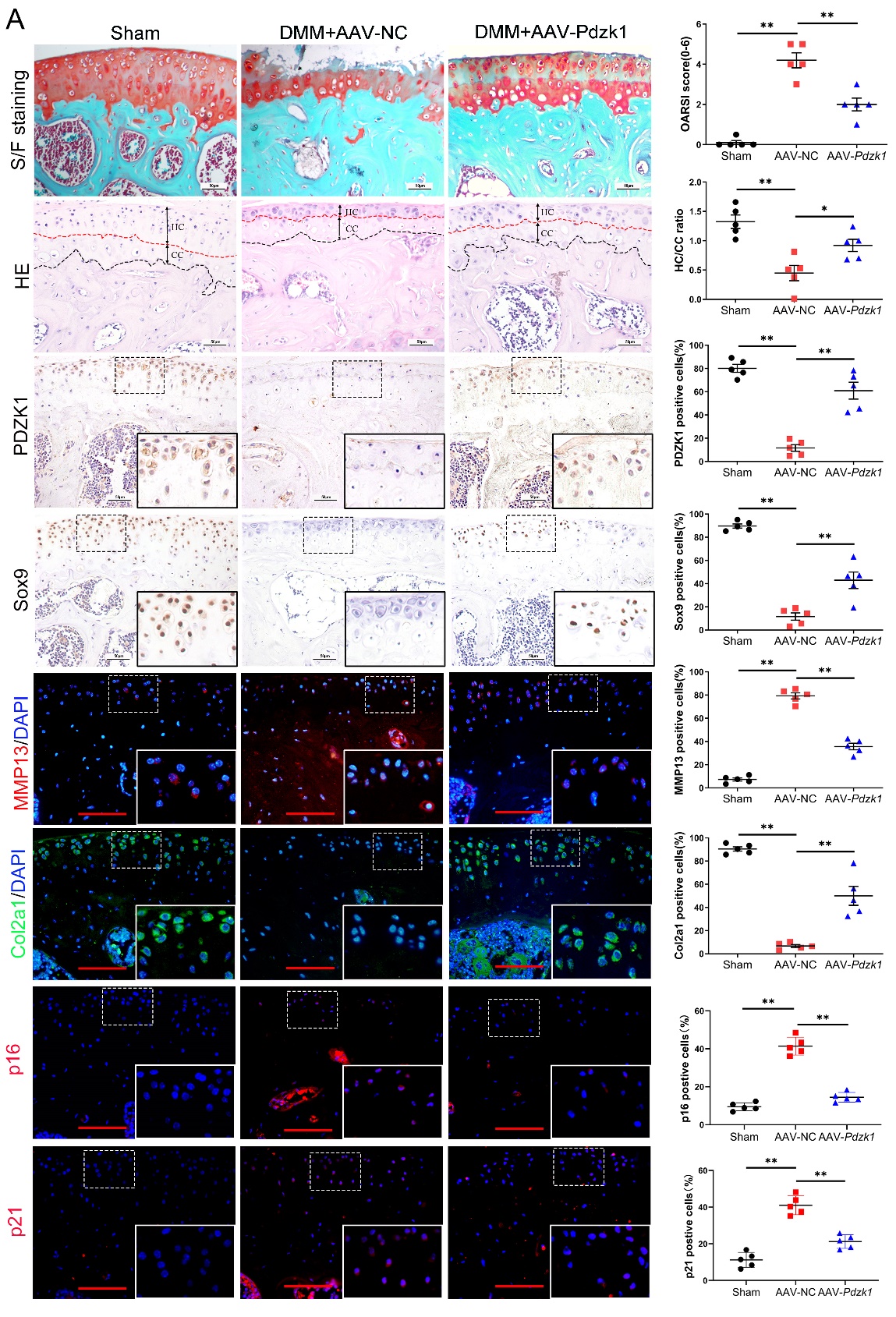


Supplementary figure 6 (A) Representative images of safranin O/fast green staining, HE staining, IHC staining of PDZK1 and SOX9 and immunofluorescence of MMP13, Col2a1, p16^INK4a^ and p21 in articular cartilage of PDZK1-expressing adeno-associated virus (AAV-Pdzk1) and control mice at 8 weeks after DMM surgery. Scale bars: 50μm. Quantitative analysis of OARSI scale, HC/CC ratio and positive chondrocytes. n=5 per group. **P<0.01.


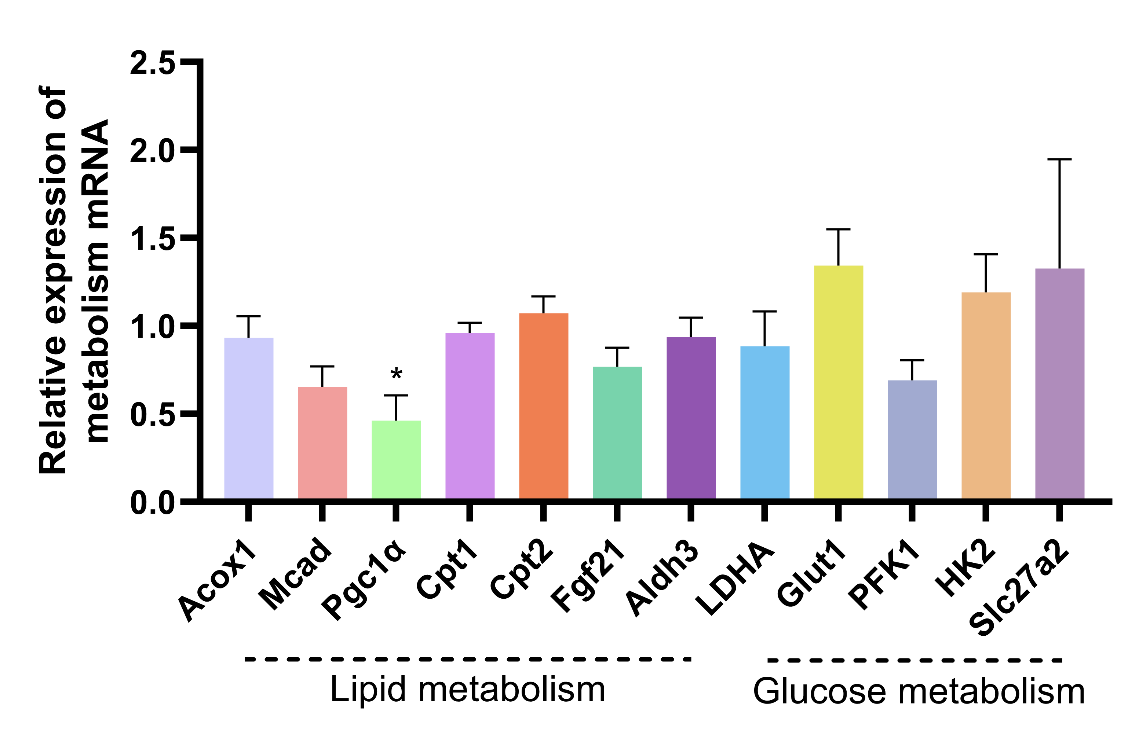


Supplementary figure 7 Rate-limiting enzymes associated with lipid and glucose metabolism in the primary chondrocytes of *Pdzk1*KO and control mice were assessed by real-time PCR. n=5 per group. *P<0.05.


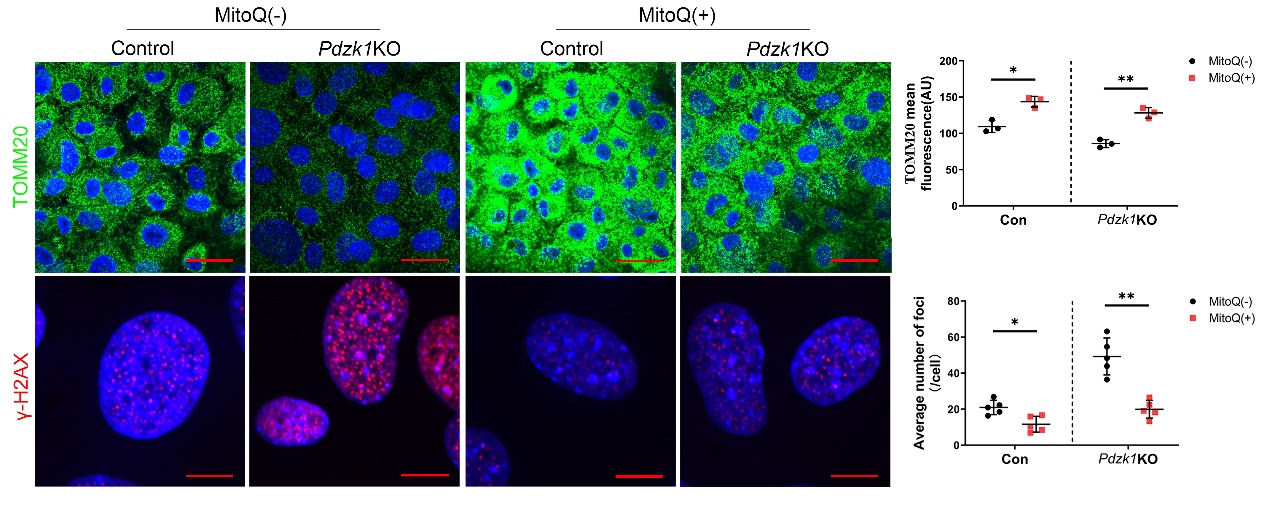


Supplementary figure 8 Representative images of immunofluorescence of TOMM20 and γH2AX of primary chondrocytes of *Pdzk1*KO and Control mice with or without MitoQ. Scale bar: 10μm (TOMM20) 5μm (γH2AX). TOMM20 mean fluorescence and average number of foci per cell were analyzed. n=3 per group (TOMM20) and n=5 per group (γH2AX). *P<0.05. **P<0.01.


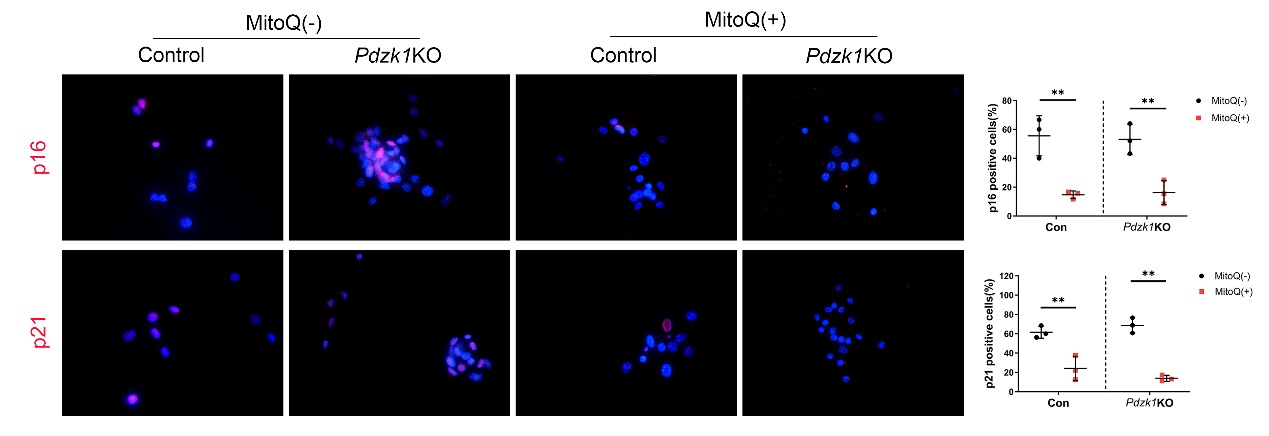


Supplementary figure 9 Representative images of immunofluorescence of p16^INK4a^ and p21 and quantification analysis of positive chondrocytes as a proportion of the 3D-cultured chondrocytes. n=3 per group. **P<0.01.


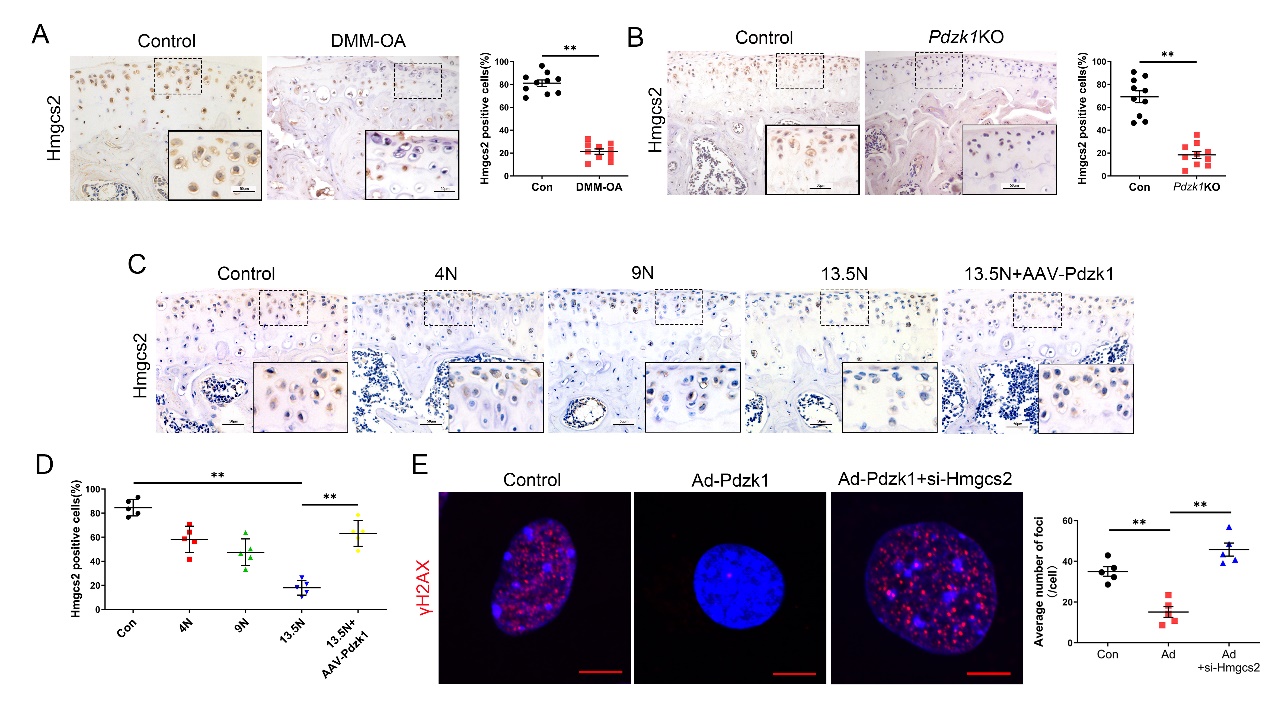


Supplementary figure 10 (A-B) Representative images of IHC staining of Hmgcs2 and quantification analysis of Hmgcs2-positive chondrocytes as a proportion of the total chondrocytes in articular cartilage of control and DMM mice. Scale bars: 50μm. n=10 per group. (C-D) Representative images of IHC of Hmgcs2 and quantitative analysis of Hmgcs2-positive chondrocytes as a proportion of the total chondrocytes in articular cartilage of mice treated with multiple loading episodes at peak loads of 4.0, 9.0 and 13.5N with or without AAV-Pdzk1. Scale bars: 10μm. n=5 per group. (E) Representative images of immunofluorescence of γH2AX of primary chondrocytes treated with Ad-PDZK1 or si-Hmgcs2. Scale bar: 5μm. Average number of foci per cell were analyzed. n=5 per group. **P<0.01.


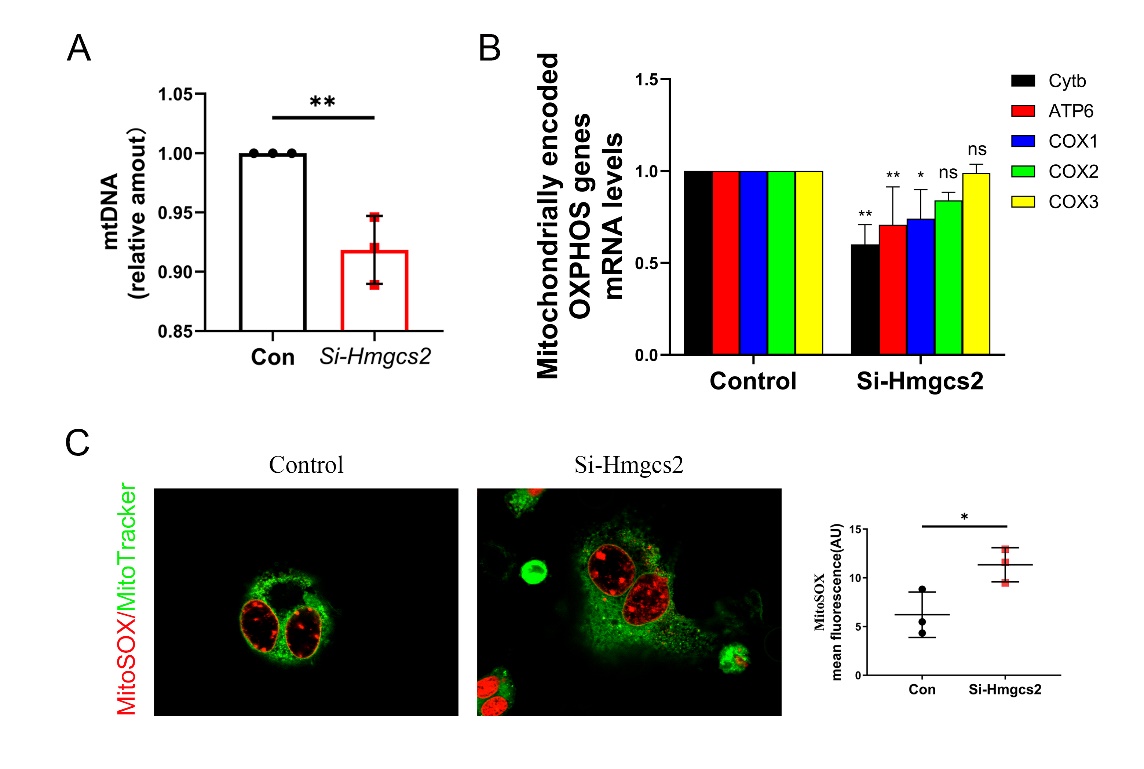


Supplementary figure 11 (A) Mitochondrial DNA (mtDNA) content in primary chondrocytes with or without si-Hmgcs2 was analyzed. n=3 per group. (B) Cytb, ATP6, COX1, COX2 and COX3 mRNA levels in primary chondrocytes with or without si-Hmgcs2 were assessed by real‐time PCR. n=3 per group. (C) Representative fluorescence of MitoTracker green and mitoSOX show mitochondria mass and ROS production of primary chondrocytes with or without si-Hmgcs2. n=3 per group. NS, not significant. *P<0.05. **P<0.01.

| Gene | Species | Primer |
| --- | --- | --- |
| *Slc9a1* | Mouse | Forward: ACCTGTTCCTCACCGCCATCAT  Reverse: GTGTGGATCTCCTCGTTGATGG |
| *Slc9a2* | Mouse | Forward: GTGTCCACTGTTGGGAAGAACC  Reverse: ACTGGTCCTTGAAGGTTAGCGG |
| *Slc9a3* | Mouse | Forward: TCTGTTTGTCAGCACCACTCTCA  Reverse: TCACGATGCTCGCTCCTCTTCA |
| *Slc9a3r1* | Mouse | Forward: ATCTGCCTCCAGCGATACCAGT  Reverse: AGCCAAGGAGATGTTGAGGTCC |
| *Slc9a3r2* | Mouse | Forward: ACTGCCATCACCAGTCACGAAC  Reverse: TCTCGCTTCCAGGTACTGCCAT |
| *Pdzk1* | Mouse | Forward: TAGAGAACGCCAGCCATGAGGA  Reverse: CAAACTGGCTGTCTCCCTCTTG |
| *Pdzd3* | Mouse | Forward: GAGTGACGCTTTCCTCTGTCCT  Reverse: CTGAGCACCAACCAGAAAGGAC |
| *Slc9a4* | Mouse | Forward: GTGGAACTGGGCTTTCATCTGC  Reverse: CTCCTCTCACACCGCTGTAGAA |
| *Cytb* | Mouse | Forward: ACAGCAAGAGCACCTGGGTGAT  Reverse: GTTCTCGTGCATCCGTAGAGTG |
| *Atp6* | Mouse | Forward: CTGTTATCCTCGGCATCATCCAC  Reverse: CAGGTAGCCAAACAACGAGGAC |
| *Cox1* | Mouse | Forward: CCCAGATATAGCATTCCCACG  Reverse: ACTGTTCATCCTGTTCCTGC |
| *Cox2* | Mouse | Forward: GCGACATACTCAAGCAGGAGCA  Reverse: AGTGGTAACCGCTCAGGTGTTG |
| *Cox3* | Mouse | Forward: CGTGAAGGAACCTACCAAGG  Reverse: CGCTCAGAAGAATCCTGCAA |
| *Timm8a2* | Mouse | Forward: GCAGTTGCTCATCCACCACATG  Reverse: CTGGCTCGTATCAATGAAGCGC |
| *Slc25a2* | Mouse | Forward: CTGCTTCCTGAAGACATACGCC  Reverse: CACTTTCCTGACAAACTGCTGGC |
| *Nptx1* | Mouse | Forward: TACACCAACGGATCAGCGAGCT  Reverse: GGTGAAGGCATACATCTCTGGC |
| *Hmgcs2* | Mouse | Forward: GGTGTCCCGTCTAATGGAGA  Reverse: ACACCCAGGATTCACAGAGG |
| *Star* | Mouse | Forward: GTGCTTCATCCACTGGCTGGAA  Reverse: GTCTGCGATAGGACCTGGTTGA |
| *Gapdh* | Mouse | Forward: CATCACTGCCACCCAGAAGACTG  Reverse: ATGCCAGTGAGCTTCCCGTTCAG |
| MtDNA*-Rps18* | Mouse | Forward: TGTGTTAGGGGACTGGTGGACA  Reverse: CATCACCCACTTACCCCCAAAA |
| MtDNA*-Cox2* | Mouse | Forward: ATAACCGAGTCGTTCTGCCAAT  Reverse: TTTCAGAGCATTGGCCATAGAA |

**Table S1 Gene qPCR primer**
